# Low Dose of Fluoride in the Culture Medium of *Cordyceps militaris* Promotes Its Growth and Enhances Bioactives with Antioxidant and Anticancer Properties

**DOI:** 10.3390/jof7050342

**Published:** 2021-04-28

**Authors:** Xiaoshuai Li, Jia Wang, Huayue Zhang, Long Xiao, Zhongfang Lei, Sunil C. Kaul, Renu Wadhwa, Zhenya Zhang

**Affiliations:** 1 Graduate School of Life & Environmental Science, University of Tsukuba, Ibaraki 305-8575, Japan; li.xiaoshuai@aist.go.jp (X.L.); wang-jia0819@aist.go.jp (J.W.); zhang-huayue@aist.go.jp (H.Z.); s1830290@u.tsukuba.ac.jp (L.X.); lei.zhongfang.gu@u.tsukuba.ac.jp (Z.L.); 2 AIST-INDIA DAILAB, National Institute of Advanced Industrial Science & Technology (AIST), Tsukuba, Ibaraki 305-8565, Japan; s-kaul@aist.go.jp

**Keywords:** *Cordyceps militaris*, potassium fluoride, enhanced bioactives, antioxidant, anticancer

## Abstract

*Cordyceps militaris* possesses several compounds with medicinal properties, and is commonly used in traditional Chinese functional food and medicine for a variety of health benefits. Because of its rare occurrence in nature, the market demand for artificial *C. militaris* is on the rise. Furthermore, efforts to increase its bioactive ingredients have also been considered in research. In this study, we aimed to investigate the effect of fluoride on the growth and enrichment of bioactive compounds in *C. militaris*. A wide range of potassium fluoride concentrations (0, 0.001, 0.01, 0.1, and 1 mM) were added to the culture media as a source of fluoride during the cultivation of *C. militaris* fruiting bodies. The contents of fluorine and bioactive substances of the fruiting bodies in normal (NM) and fluorine-supplemented (FM) media were measured and compared. *C. militaris* raised in *the* growth medium supplemented with 0.01 mM potassium fluoride led to a 44.86% (1.55 ± 0.14 g/bottle) increase in biomass and a 23.43% (3161.38 ± 35.71 µg/g) increase in total carotenoid content in the fruiting bodies. Furthermore, a remarkable increase in superoxide dismutase-like activity (84.75 U/mg) and 2,2-diphenyl-1-picrylhydrazyl radical scavenging activity (IC_50_ = 2.59 mg/mL) was recorded. In human cancer cell-based assays, *C. militaris* raised in FM caused stronger cytotoxicity, apoptosis, and cell cycle arrest in human osteosarcoma cells. These results demonstrated that a low dose of fluoride could stimulate the growth of *C. militaris* fruiting bodies and enhance the production of bioactive ingredients that possess useful antioxidant and anticancer activities.

## 1. Introduction

*Cordyceps militaris* is a traditional Chinese entomopathogenic fungus that has long been used as an alternative medicine due to its antioxidative, anti-inflammatory, antibacterial, and anticancer activities [1,2,3]. The beneficial effects of *C. militaris* are attributed to the presence of abundant biologically active substances, including cordycepin, cordyceps acid, cordyceps polysaccharides, ergosterol, and mannitol [4]. It also possesses considerable amounts of carotenoids, the phytopigments that serve as light harvesting and photoprotective agents in the process of photosynthesis in the plant. For human, carotenoids serve as an important bioactive resource for antioxidants and vitamins that are essential for various physiological functions. Carotenoids have also been shown to possess various disease-preventive and therapeutic activities [5,6,7,8,9]. However, the resource of this wild edible fungus has been decreasing sharply in the recent past and has been reported to be insufficient to meet the huge market demand [10,11]. In view of this, production of *C. militaris* in a controlled environment has been initiated, which involves various technologies including the development of temperature-controlled microfluid devices and the use of biopolymers [12,13]. At the same time, approaches to optimize the artificial culture medium (content/ratio of carbon and nitrogen sources or adding trace elements such as selenium) [14,15,16] to promote the production of bioactive compounds in the *C. militaris* fruiting bodies have also been adopted. Growth conditions have been shown to affect the production of metabolites and hence to obtain a uniform and stable content, production under controlled conditions is favorably considered. Cultivation in a controlled environment is also important for the protection of *Cordyceps* in the wild. In light of these premises, several studies in the past have focused on optimization of the culture conditions [17] and media composition [18] for mycelia liquid culture. However, only a few studies have investigated the conditions for solid-state cultivation of fruiting bodies.

Fluoride is widespread in the environment; occurs in the air, water, soil, and plants; and is also an essential element for animals and humans [19]. As the most electronegative element, fluorine has important applications in various fields [20], including industry, agriculture, and medicine [21,22]. Although a continuous excessive amount of fluoride ingestion may lead to fluorosis and have harmful effects on organisms [23,24], many notable discoveries related to positive stimulation at low doses of fluoride have also been reported in *Pinus* [25], algae [26], animals [27], and silkworms [28]. Regarding this phenomenon, Burgstachlert proposed that fluoride possesses a hormesis effect [29], a dose–response activity in which opposite responses are observed at low and high doses for the same measured parameter [30]. In view of these reports, we set out to evaluate the effect of fluoride on the growth and bioactivity of *C. militaris*.

In the present study, potassium fluoride (KF) was added as a fluoride source to the culture media. To the best of our knowledge, this is the first study to investigate the effect of fluoride on *C. militaris* growth and enhancement of bioactive compounds in its fruiting bodies in normal and KF-supplemented media. Furthermore, the anticancer activity of their extracts was compared using human cancer cells in vitro.

## 2. Materials and Methods

### 2.1. Chemicals and Reagents

The *C. militaris* strain KCTC 6064 was purchased from the Wuhan Academy of Agricultural Sciences in China (Wuhan, China). Glucose, peptone, KH_2_PO_4_, MgSO_4_, KF, and acetone were purchased from FUJIFILM Wako Pure Chemical Corporation (Osaka, Japan). Rice was purchased from the National Federation of Agricultural Co-operative Associations (Ibaraki, Japan). Superoxide dismutase (SOD) Assay Kit-WST was purchased from Dojindo Molecular Technologies, Inc. (Tabaru, Kumamoto, Japan); 2,2-diphenyl-1-picrylhydrazyl (DPPH), Dulbecco’s Modified Eagle Medium (DMEM), fetal bovine serum (FBS), and the penicillin–streptomycin solution were purchased from Thermo Fisher Scientific K.K. (Tokyo, Japan).

### 2.2. Cell Lines

Human osteosarcoma (U2OS) and normal lung fibroblasts (TIG-3) were purchased from the National Institute of Physical and Chemical Research (RIKEN, Japan) and the Japanese Collection of Research Bioresources (JCRB) Cell Bank, respectively. Cells were cultured in DMEM containing 10% FBS supplemented with 1% penicillin–streptomycin at 37 °C and 5% CO_2_. Typically, cells were plated and incubated for 24 h to reach a stable adherence status and then used for experiments.

### 2.3. Cordyceps militaris Cultivation

The stock culture of *C. militaris* strain KCTC 6064 was maintained on agar slants containing 2% glucose, 2% peptone, 0.2% KH_2_PO_4_, and 0.3% MgSO_4_. The inoculated slants were incubated at 21–23 °C in the dark for 10 days and then stored at 4 °C for mycelial growth. The normal solid medium comprised 36 g of rice with 63 mL of the nutrient solution (2% glucose, 2% peptone, 0.2% KH_2_PO_4_, and 0.3% MgSO_4_) in a 300 mL cylindrical glass bottle. The KF content added to the nutrient solution was adjusted to 0.001, 0.01, 0.1, and 1 mM, to set different concentrations of KF-supplemented media. All media were then sterilized for the cultivation of *C. militaris* on a solid medium. Each solid medium was prepared for 5 parallel bottles. After the completion of mycelial formation, they were transferred to normal and KF-supplemented solid culture media and incubated in the cultivation shed, where the temperature was maintained at 21–23 °C with the air humidity above 70% and in the dark for 20 days for base cultivation. After the complete spread of *C. militaris* mycelia on the medium surface, they were subjected to an alternating light–dark cycle of 21–23 °C in 12 h of light and 16–20 °C in 12 h of darkness for 30 days to stimulate fruiting body growth. The cultivation environment and growth status were examined regularly. After 30 days, *C. militaris* fruiting bodies from normal (NM) and fluorinated media (FM; 0.001 to 1 mM)) were harvested, vacuum freeze-dried, weighed, collected, ground into powder, and stored at −20 °C for further analysis.

### 2.4. Fluoride Detection and Quantification

^19^F nuclear magnetic resonance (NMR) spectra of *C. militaris* grown in NM and FM were recorded on a Bruker AVANCE 400 (400 MHz) NMR (PS751) (Bruker Corporation, Japan) with trichlorofluoromethane (CFC-11) (0.5 wt %) as an internal standard in MeOD-d4 at 25 °C.

Fluorine was quantitated using ^19^F-NMR and compared with 1,1,1,3,3,3-hexafluoroisopropanol (HFip) as follows:

(1) The sample m (*x*)g and the internal standard 1,1,1,3,3,3-hexafluoroisopropanol n(std) mol were mixed in 1 mL of MeOD-d_4_.
(2) The above mixture (600 μL) was analyzed using ^19^F NMR, and the ratio of the area of the sample to the internal standard was obtained, which was equal to the molar ratio of fluorine in the sample and 1,1,1,3,3,3-hexafluoroisopropanol: Area(std)/Area(*x*) = 6 × *n*(std)/*n*(*x*)
(3) The quantification of fluorine in *C. militaris* was estimated as follows: 19 × *n*(*x*)/*m*(*x*) × 100%

### 2.5. Carotenoid Quantification

To each group of *C. militaris* fruiting body powder (1.0 g), 20 mL of acetone was added and mixed evenly. The mixture was then heated in a microwave oven at 200 W for 3 min. The samples were shaken at 25 °C for 30 min and centrifuged at 2500× *g* for 15 min. The supernatant was collected and diluted with acetone. The absorbance was measured at 475 nm using a spectrophotometer (Lambda35, Perkin Elmer Co. Ltd., Akron, OH, USA). The total carotenoid content (TCC) was calculated using the following equation [31]:

TCC (µg/g) = As × V × D/(0.16 × W) (1)

where *As* is the absorbance of each sample, *V* is the dosage of acetone (mL), *D* is the dilution factor, 0.16 is the molar extinction coefficient of carotenoids, and *W* is the mass of *C. militaris* powder (g).

### 2.6. Preparation of Aqueous Extracts from C. militaris Fruiting Bodies

The *C. militaris* fruiting body powder (2.0 g) was extracted with 40 mL deionized water at 100 °C for 45 min, and the process was repeated thrice. After cooling down to 25 °C, all the aqueous extracts were collected and centrifuged at 5000× *g* at 25 °C for 15 min. Each supernatant was filtered through a 0.2 µm sterile filter membrane and concentrated in a rotary evaporator at 60 °C. Finally, the concentrated solutions were lyophilized to obtain a powder. These aqueous extract powders prepared from *C. militaris* grown in NM and FM were stored at −20 °C for further analysis.

### 2.7. Antioxidant Activity Assay

#### 2.7.1. Assay for SOD-Like Activity

The SOD-like activity of lyophilized aqueous extracts of *C. militaris* grown in NM or FM was measured using the SOD Assay Kit-WST, following the manufacturer’s instructions. Briefly, the WST working solution with the enzyme standard was allowed to react with various concentration samples, and the absorbance changed with decreasing enzyme concentrations. Therefore, SOD-like activity (inhibition rate, %) was determined by measuring the absorbance of each sample.

#### 2.7.2. DPPH Radical-Scavenging Activity Assay

The DPPH (2,2-diphenyl-1-picrylhydrazyl) radical-scavenging activity of the aqueous extract samples was measured following the method described by Yuan, with some modifications [32]. Different concentrations (0.016, 0.08, 0.4, 2, and 10 mg/mL) of *C. militaris* extracts were mixed with a methanol solution of DPPH (1:3, *v*/*v*). After reaction at 25 °C in the dark for 30 min, the optical density (OD) of the samples and the blank was measured. A decrease in the absorbance of the DPPH solution indicated the promotion of DPPH radical-scavenging activity.

### 2.8. Anticancer Activity Assays

#### 2.8.1. Cell Viability Assay

TIG-3 and U2OS cells were seeded in 96-well plates at a density of 5000 cells/well and incubated at 37 °C and 5% CO_2_. After 24 h of incubation, the cells were cultured in varying concentrations of extracts prepared from *C. militaris* maintained in either NM or FM for 24 h. Cell viability was measured by the MTT (3-(4,5-dimethylthiazol-2-yl)-2,5-diphenyltetrazolium bromide; a tetrazolium dye) assay. MTT was then added into each well, and the samples were incubated at 37 °C for 4 h. The medium in each well was replaced with 100 µL of dimethyl sulfoxide to dissolve the insoluble purple formazan generated from yellow MTT by living cells. The optical density (OD) of the wells was measured at 570 nm using a microplate reader (Infinite M200 PRO, TECAN, Männedorf, Switzerland).

#### 2.8.2. Apoptosis Assay

Apoptosis was determined by Annexin-V and 7-aminoactinomtcin (7-ADD) double staining. U2OS cells (2.0 × 10^5^/well) were seeded in 6-well plates and incubated for 24 h and then treated with extracts of *C. militaris* fruiting bodies obtained from cultures in NM and FM (0.001 mM) at 0.25 mg/mL for another 24 h. Cells were harvested and rinsed with phosphate-buffered saline (PBS) and stained with 100 µL of Guava Nexin Reagent (Millipore) and incubated at room temperature (23–25 °C) in the dark for 40 min. The apoptotic cells were estimated with a flow cytometer (Guava PCA-96, Millipore, Billerica, MA, USA).

#### 2.8.3. Cell Cycle Assay

U2OS cells (2.0 × 10^5^ cells/well) were seeded in 6-well plates and incubated for 24 h, followed by treatment with *C. militaris* supplemented with NM and FM at 0.125 mg/mL for another 24 h. Cells were suspended in 70% ethanol and incubated at −20 °C for 24 h, then collected by centrifugation at 500× *g* at 4 °C for 5 min. Ethanol was removed and the cell suspension was washed with PBS and centrifuged to collect the cell pellet, which was re-suspended in 1 mL cold PBS (containing RNAse-of 100 µg/mL). Pellets were incubated at 37 °C for 2 h, followed by centrifugation and the addition of 200 µL Guava Cell Cycle Reagent. The number of cells in each stage of the cell cycles was estimated by flow cytometry (Guava PCA-96, Millipore, Billerica, MA, USA).

#### 2.8.4. Western Blotting Assay

Cancer cells (2.0 × 10^5^ cells/well) were seeded in 6-well plates, incubated for 24 h, and then treated with *C. militaris* extracts (0.25 mg/mL for 24 h). Cells were harvested and lysed in a RIPA (radioimmunoprecipitation assay) buffer (Thermo Fisher Scientific, Waltham, MA, USA) supplemented with a protease inhibitor cocktail (Roche Applied Science, Mannheim, Germany). Cell lysates with 20 µg of protein was resolved in SDS polyacrylamide gel and transferred to the polyvinylidene fluoride (PVD) membrane by a semi-dry transfer apparatus (ATTO Corporation, Tokyo, Japan) for 75 min. The membrane was blocked with 3% bovine serum albumin (BSA) for 1 h, followed by incubation with primary antibodies overnight at 4 °C. The membrane was washed with Triton X-100 Tris-buffered saline (TTBS) thrice (10 min each) and finally incubated with secondary specific antibodies at room temperature for 1 h. The membranes were then washed with TTBS and the signals were detected with an enhanced chemiluminescent (ECL) kit (Thermo Scientific, Waltham, MA, USA). The quantification of relative expression for each protein was determined through Image J 1.53 software.

### 2.9. Statistical Analysis

All the assays were performed in triplicate, and the results were expressed as means ± standard deviation (SD). The degree of statistical significance between the control and sample groups was analyzed using an unpaired *t*-test (GraphPad Prism 6 software, San Diego, CA, USA). Significant values are represented as * *p* < 0.05, **p < 0.01, and *** *p* < 0.001.

## 3. Results and Discussion

### 3.1. Dry Weight and Total Carotenoid Content of C. militaris Raised in the Normal (NM) and Flouride-Supplemented (FM) Medium

**Figure 1:**
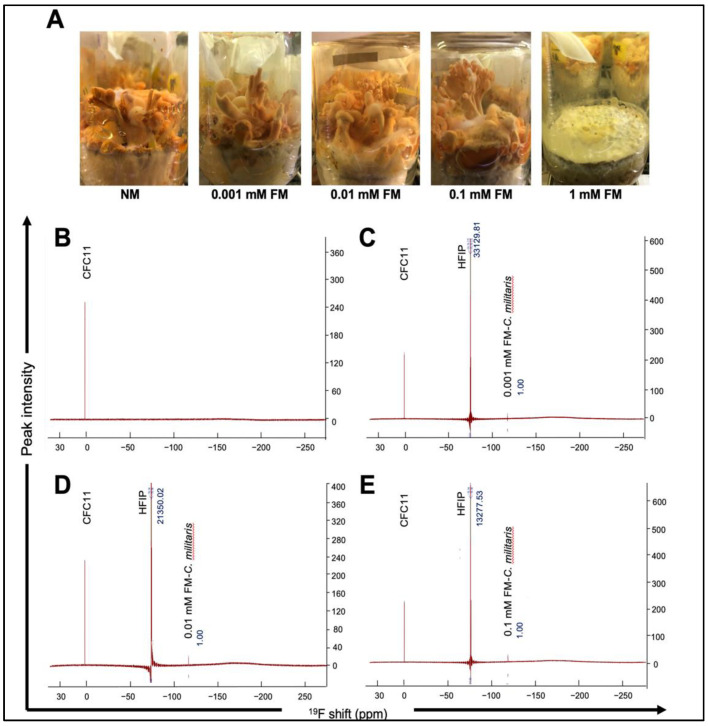
(**A**) Fruiting bodies of *C. militaris* cultured in normal and fluoride-supplemented media are shown. ^19^F NMR spectra of fruiting bodies raised in NM-*C. militaris* (**B**), 0.001 mM FM *C. militaris* (**C**), 0.01 mM FM *C. militaris* (**D**), and 0.01 mM FM *C. militaris* (**E**).

In the present study, we successfully obtained *C. militaris* fruiting bodies from normal and potassium fluoride-supplemented media (0.001, 0.01, and 0.1 mM). As shown in Figure 1A, we found that in the 1 mM FM group, the growth process completely stopped after mycelia covered the surface of the solid medium in all five bottles (one each for normal as well as fluoride-supplemented media) examined. This was in line with earlier studies that have reported the inhibitory effect of high doses of fluoride on the growth of organisms [33]. Notably, as shown in Table 1, 1.55 ± 0.14 g/bottle dry weight of fruiting bodies was obtained from 0.01 mM FM. The mass of freeze-dried fruiting bodies from NM, 0.001 mM FM_,_ and 0.1 mM FM was 1.07 ± 0.07, 1.28 ± 0.13, and 1.06 ± 0.12 g/bottle, respectively. We found that the biomass of *C. militaris* fruiting bodies was promoted in the solid-state medium containing a low dose of fluoride. The optimization of fluoride addition (0.01 mM) resulted in an increase in fruiting bodies to 44.86% (1.55 ± 0.14 g/bottle). We next analyzed the total carotenoid content (TCC) in *C. militaris* cultured in NM and FM. Carotenoids are important natural pigments with many bioactive functions, including antioxidation, anti-inflammation and anticancer activity [34], and are commonly found in plants, fungi, and algae [35]. *C. militaris* is a well-known potential source of natural carotenoids [36]. As one of the primary active substances, the TCC of *C. militaris* was evaluated. As shown in Table 1, the TCC of *C. militaris* raised in NM, 0.001, 0.01, and 0.1 mM FM was 2561.27 ± 26.56, 2751.00 ± 21.36, 3161.38 ± 35.71, and 3035.96 ± 53.30 µg/g, respectively. The TCC of the 0.01 mM FM group increased by about 23.43% as compared with the NM group. This was better than *Rhodotorula glutinis*, a fungal elicitor, which stimulated the accumulation of carotenoids by 13.72% [37]. According to a previous study, the TCC of *C. militaris* fruiting bodies was significantly increased through stimulation by light [38]. On the other hand, low concentrations of fluoride having no effect [39] or positively stimulating photosynthetic pigments have also been reported [25]. In the present study, we found that carotenoid accumulation in *C. militaris* was enhanced at low doses of fluoride.

### 3.2. ^19^F NMR Spectra of NM and FM Raised C. militaris

Fluoride was commonly accumulated in plants through airborne deposition or direct uptake from the soil [40,41]. Despite the success of fluorine in the design of synthetic bioactive compounds, nature appears to have evolved only a limited set of biogenic organofluorides, like the fluoroacetate discovered in the leaves of the South African plant *Dichapetalum cymosum* [42]. A possible explanation for the limitation of biologically produced organofluorides is the finite bioavailability of fluorine [43]. In order to determine whether fluorine participates in metabolic processes in *C. militaris* fruiting bodies and to identify the form in which fluorine existed, we detected and quantified the status of fluorine by ^19^F NMR spectra. As shown in Figure 1B, there was no fluoride signal in NM *C. militaris*. The results for 0.001, 0.01 and 0.1 mM FM are shown in Figure 1C–E, respectively; the three peaks (from left to right) represent the internal standard of CFC-11, the quantitation standard of 1,1,1,3,3,3-HFip, and the fluorine signal of each sample. We observed that the strength of the fluorine signal in FM *C. militaris* gradually increased from 0.001 mM to 0.1 mM by comparing the peak value with that of 1,1,1,3,3,3-HFip. The deviation value between FM *C. militaris* (approximately 118 ppm) and the internal standard CFC-11 (0 ppm) was very close to the deviation value of KF [44]. Therefore, we assumed that the fluoride detected in FM *C. militaris* existed as inorganic ions.

The quantification of total fluoride content (TFC) is summarized in Table 2. The TFC in the extracts of NM, 0.001, 0.01, and 0.1 mM FM *C. militaris* groups were 0, 15.09, 33.81, and 54.38 ppm, respectively. For fluorosis risk evaluation, according to Huimei Cai’s reports of Chinese tea, a beverage with a similar cooking method to *C. militaris*, had an average fluoride concentration of 81.7 ppm in infusions of 115 commercially available teas from Chinese tea markets, which was much higher than the accumulated fluoride in this study. The data indicate that there is no risk of fluorosis as reported earlier [45]. Furthermore, organic fluorine signals were undetectable, indicating that the fluoride ions were unlikely to participate in the synthesis of organic active substances but acted in the inorganic form in the metabolism of *C. militaris*.

### 3.3. SOD-Like Activity Assay

**Figure 2:**
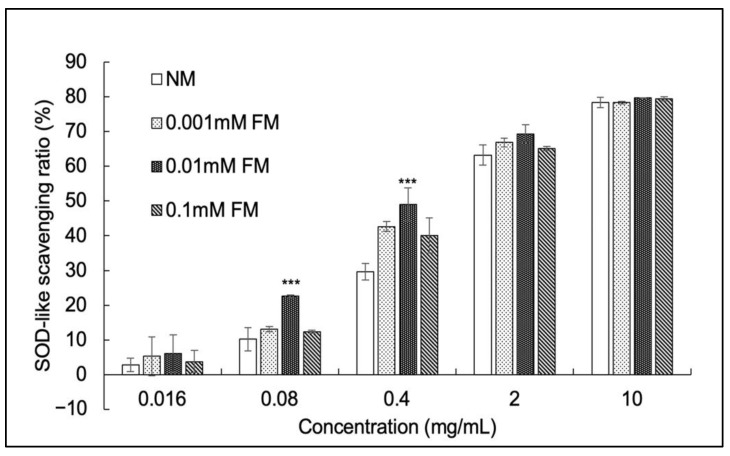
Superoxide dismutase-like activity of aqueous extracts (*x*-axis) of fruiting bodies obtained from *C. militaris* cultured in media supplemented with the indicated concentrations of fluoride (mean ± SD, *n* = 3), *** *p* < 0.001 (Student’s *t*-test vs. NM).

Superoxide dismutase (SOD) is a metalloenzyme that catalyzes the transformation of superoxide into oxygen and hydrogen peroxide. It functions in the immune response of organisms and in defense against toxic superoxide radicals [46]. The SOD-like activity assay is widely used to evaluate antioxidant capability. As shown in Figure 2, the SOD-like activity in all samples constantly increased in a dose-dependent manner. The SOD-like scavenging ratio of 0.01 mM FM *C. militaris* was significantly higher than that of NM *C. militaris* at the range of 0.08 to 0.4 mg/mL. The half maximal inhibitory concentration (IC_50_) values (Table 3) of NM and FM *C. militaris* at 0.001, 0.01 and 0.1 mM were 1.13, 0.81, 0.59, and 0.87 mg/mL, respectively. The IC_50_ of SOD in NM *C. militaris* (1.13 mg/mL) is very close to previously reported results on *C. militaris* (SOD IC_50_ around 1 mg/mL) [47]. The SOD activity (one unit of SOD is defined as the amount of the enzyme in the sample solution that inhibits the reduction reaction of WST-1 with superoxide anions by 50%) of NM *C. militaris* (44.25 U/mg), 0.001 mM FM *C. militaris* (61.37 U/mg), 0.01 mM FM *C. militaris* (84.75 U/mg), and 0.1 mM FM *C. militaris* (57.47 U/mg) was also calculated for evaluation. There are several reports on the effect of fluoride stress on SOD activity in various organs of plants [48] or animals [49]. However, in the present study, we found that the SOD-like activity of 0.01 mM FM *C. militaris* increased remarkably (84.75 U/mg). A similar enhancement of SOD activity in edible crops of India with increasing concentrations of fluoride has also been reported by Chakrabarti [50]. The fluoride stimulation possibly causes an increase in SOD activity through enhanced SOD biosynthesis or metabolism activities as a positive feedback mechanism [51].

### 3.4. DPPH Radical Scavenging Activity Assay

**Figure 3:**
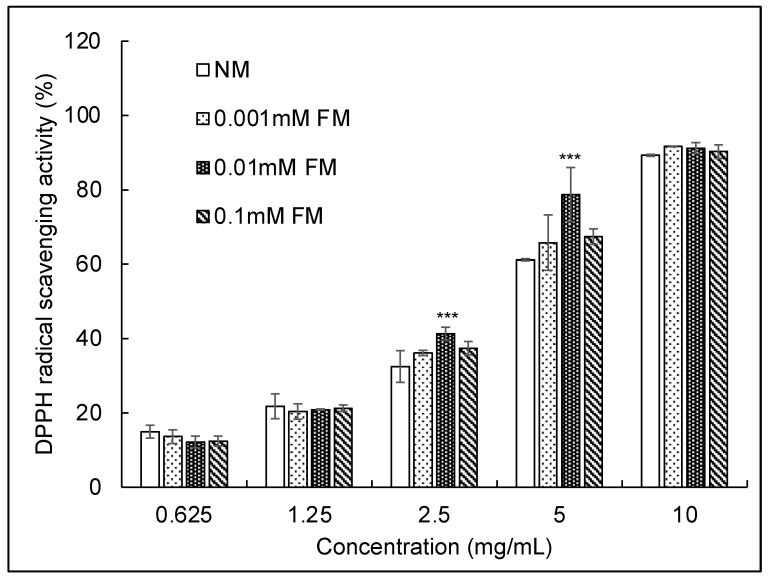
The 2,2-diphenyl-1-picrylhydrazyl radical scavenging activities of aqueous extracts (*x*-axis) of fruiting bodies obtained from *C. militaris* cultured in media supplemented with the indicated concentrations of fluoride, (mean ± SD, *n* = 3), *** *p* < 0.001 (Student’s *t*-test vs. NM).

Figure 3 shows the DPPH radical scavenging activities of the four samples. All samples showed a dose-dependent inhibition of DPPH radical activity. The scavenging ratios of samples at 10 mg/mL of DPPH radicals were 89.32, 91.66, 91.20, and 90.30 for NM, 0.001 mM FM, 0.01 mM FM, and 0.1 mM FM *C. militaris*, respectively. Notably, 0.01 mM FM *C. militaris* exhibited the strongest DPPH radical scavenging activity from 2.5 to 5 mg/mL. The IC_50_ value (Table 3) of DPPH for NM *C. militaris* (4.16 mg/mL) was consistent with an earlier report on *C. militaris* (4.62 mg/mL) [50]. The IC_50_ values for 0.001 mM, 0.01 mM, and 0.1 mM FM *C. militaris* were 2.92 mg/mL, 2.59 mg/mL, and 2.89 mg/mL, respectively, and 0.01 mM FM *C. militaris* exhibited conspicuous free radical scavenging ability. This finding, combined with the results for SOD-like scavenging activity, suggests that the antioxidant ability of *C. militaris* was significantly improved by FM.

### 3.5. FM-C. militaris Extracts Showed Enhanced Cytotoxicity in Cancer Cells

**Figure 4:**
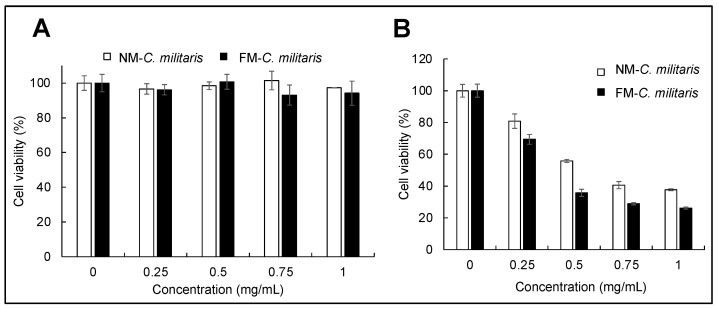
MTT assay of the normal TIG-3 cells (**A**) and the cancerous U2OS (**B**) cell line treated with aqueous extracts (*x*-axis) of fruiting bodies obtained from *C. militaris* cultured either in normal or fluoride (0.01 mM)-supplemented media for 24 h, (mean ± SD, *n* = 3).

There have been many reports describing that *C. militaris* extracts exhibit anti-proliferative effects in different cancer cells, including HeLa, A549, HT1080, and U2OS [32,47]. In this context, we further investigated the anticancer activity of *C. militaris* cultured in NM and FM media. Based on the results of the growth evaluation and antioxidant activity assays, we selected NM and 0.01 mM FM *C. militaris* as NM *C. militaris* and FM *C. militaris* to investigate their anticancer activity in U2OS cells. As shown in Figure 4A, FM *C. militaris* extract exhibited a dose-dependent and stronger anti-proliferative effect on U2OS cells than NM *C. militaris*. Figure 4B shows the cytotoxicity effects of NM and FM *C. militaris* on normal fibroblasts (TIG-3) cells. The viability of all the treated TIG-3 cells was maintained, even at a high level, indicating that there were no significant cytotoxic effects of the extracts prepared from NM or FM *C. militaris*. Cumulatively, these results demonstrated that FM *C. militaris* exerted enhanced selective cancer-cell-killing activity.

### 3.6. FM-C. militaris Extracts Caused Enhanced Apoptosis and Cell Cycle Arrest in Cancer Cells

**Figure 5:**
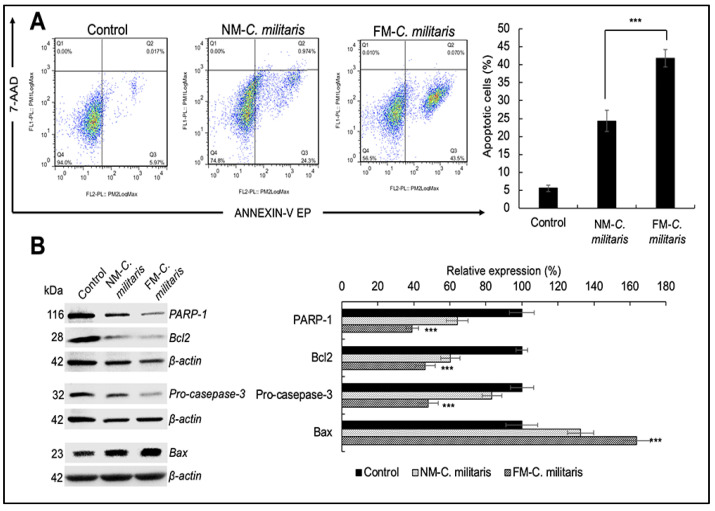
Apoptosis assay of U2OS cells treated with NM and FM *C. militaris* for 24 h. (**A**) Apoptosis analysis from flow cytometry. Quantitation of the results is shown on the right (mean ± SD, *n* = 3), *** *p* < 0.001 (Student’s *t*-test vs. NM *C. militaris*). (**B**) Western blot analysis for apoptotic proteins (PARP-1, pro-caspase 3, Bcl-2, and Bax) after 24 h. Quantitation of the results is shown on the right (mean ± SD, *n* = 3), *** *p* < 0.001 (Student’s *t*-test vs. control).

The MTT results revealed that the FM *C. militaris* exhibited stronger cytotoxicity in the U2OS cell line. For further confirmation, we checked the difference in apoptotic activity in U2OS cells. In the apoptotic cell analysis, we used Annexin V-/7-ADD-, Annexin V+/7-ADD+, and Annexin V+/7-ADD+ to represent healthy, early apoptotic, late apoptotic, and debris cells, respectively. Annexin V-positive cells (early and late apoptosis) were considered as the apoptotic cells. U2OS cells treated with either NM or FM *C. militaris* (24 h) were subjected to molecular analyses, including cell cycle and apoptosis signaling. As shown in Figure 5A, flow cytometry analysis showed that U2OS cells were concentrated in the early apoptosis stage. According to the quantitation results, we found that FM *C. militaris* induced stronger apoptosis (41.86%) in U2OS compared with NM *C. militaris* (24.36%). In order to determine the molecular mechanism of apoptosis, we analyzed the expression of the proteins involved in apoptosis. These included polyADP-ribose polymerase-1 (PARP-1), caspase 3, B-cell lymphoma 2 (Bcl2), and Bax, which are tightly involved in the apoptotic signaling pathway. Western blot analysis revealed significant downregulation of anti-apoptotic factors including PARP-1 and Bcl2 (Figure 5B). While the expression level of the pro-apoptosis marker Bax was upregulated to a large extent, the caspase family could accelerate apoptosis. There was also a significant downregulation of pro-caspase 3, indicating an increase in cleaved-caspase 3, which means that the acceleration process was activated. The quantitation data showed that FM *C. militaris* caused enhanced inhibition of the anti-apoptotic proteins, including PARP-1 (39.05%) and Bcl2 (46.23%), compared with NM *C. militaris* (64.06% for PARP-1 and 60.82% for Bcl2). In addition, the relative expression of pro-caspase 3 in cells treated with FM *C. militaris* treated (47.85%) was lower than in cells treated with NM *C. militaris* (83.31%). These data indicate that the activity of the caspase family was enhanced in cells treated with FM *C. militaris* extract. Furthermore, the relative expression of the pro-apoptotic marker Bax was significantly enhanced in cells treated with FM *C. militaris* extract (163.77%) compared with NM *C. militaris* (132.65%). These results supported the stronger induction of apoptosis in U2OS cells by the FM *C. militaris* extract treatment, which was induced through activation of Bax and caspase 3, and inhibition of PARP-1 and Bcl2.

**Figure 6:**
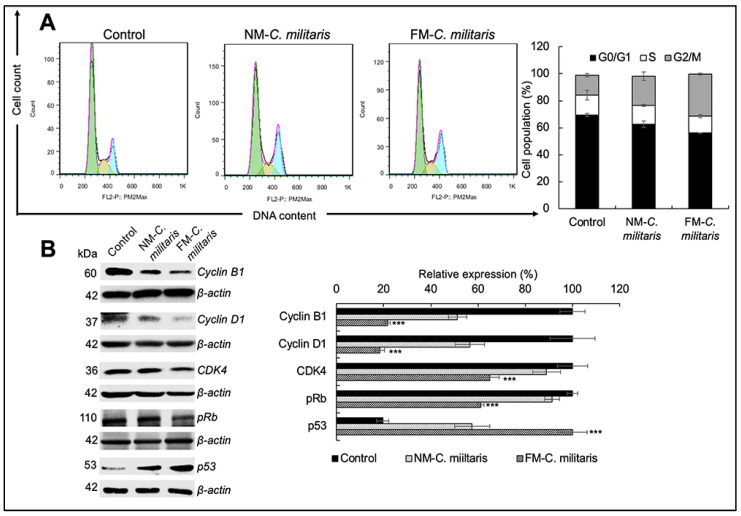
Cell cycle arrest induced by NM and FM *C. militaris* for 24 h. (**A**) Cell cycle analysis from flow cytometry. Quantitation of the results is shown on the right. (**B**) Western blot analysis for cell cycle regulatory proteins (cyclin B1, cyclin D1, CDK4, pRb, and p53) after 24 h of incubation. Quantitation of the results is shown on the right (mean ± SD, *n* = 3), *** *p* < 0.001 (Student’s *t*-test vs. control).

We also performed cell cycle analysis in U2OS cells treated with either NM or FM *C. militaris*. Figure 6A shows that the subpopulation in the G2/M phase increased from 14.72% (control) to 21.54% in NM *C. militaris*-treated cells and 31.25% in FM *C. militaris*-treated cells. This result suggested that the potential enhancement with FM *C. militaris* caused growth arrest in U2OS cells.

In order to further investigate the molecular mechanism of FM *C. militaris*-induced growth arrest, we analyzed the expression of various proteins (p53, cyclin D1, cyclin B1, CDK4, and pRb) involved in cell cycle progression. p53, a major tumor suppressor protein, was upregulated in both NM and FM *C. militaris*-treated cells; however, it showed a higher increase in the latter (Figure 6B). The activated cyclin/CDK complexes phosphorylated and inactivated members of the retinoblastoma (Rb) protein family that induce cell cycle progress. Consistent with the upregulation of p53, there was a significant decrease in the expression of cyclin D1, cyclin B1, CDK4, and pRb. Moreover, quantitation of the relative expression (Figure 6B) revealed that the downregulation of cyclin D1 (18.32%), cyclin B1 (21.69%), CDK4 (64.86%), and pRb (12.39%) was lower in cells treated with FM *C. militaris* as compared with NM *C. militaris* (56.56% for cyclin D1, 51.22% for cyclin B1, 88.98% for CDK4, and 38.77% for pRb). These results supported the ideal that the stronger growth arrest in FM *C. militaris*-treated cells was mediated by the enhanced activation of p53 and inhibition of the pRb signaling pathways. Taken together, these results demonstrated that FM *C. militaris* exerted potent anticancer effects through the induction of cell cycle arrest and apoptosis.

In the present study, we investigated a new method to increase the production of *C. militaris* fruiting bodies through the addition of fluoride to solid-state media. The optimal strategy of fluoride addition (0.01 mM) resulted in an increase in fruiting bodies by 44.86% (1.55 ± 0.14 g/bottle) and an increase in TCC in fruiting bodies to 23.43% (3161.38 ± 35.71 µg/g). According to our investigation, fluoride in solid media most likely acts as an inorganic ion form in the metabolism process in *C. militaris*. The SOD activity of *C. militaris* with 0.01 mM FM was found to be increased to 84.75 U/mg compared with NM (44.25 U/mg). Furthermore, the antioxidant activity was significantly increased as per the DPPH radical scavenging activity (the IC_50_ for NM was 4.16 mg/mL, while that for 0.01 mM FM was 2.59 mg/mL). We supposed that the mechanism of fluoride stimulation of *C. militaris* was mainly through its effect on the interaction of the light reaction and dark respiration during the fruiting bodies’ formation stage, resulting in enhanced accumulation of carotenoids, and further accelerated the metabolism of active substances like superoxide dismutase. Other reasonable explanations for the phenomenon observed in this study could be the osmotic changes or alterations in the molecular structure of the cell membrane that occurred at low concentrations of fluoride and improved the molecular exchange capacity to promote the synthesis of some active enzymes [26]. In the present study, the molecular-level comparison of the anticancer activity of U2OS cells between NM and FM *C. militaris* revealed that FM *C. militaris* induced stronger cell apoptosis in U2OS cells, possibly due to increasing cell cycle arrest in the G2/M phase through the activation of p53 and inhibition of the pRb signaling pathways.

## 4. Conclusions

Cultivation technologies to support and empower the production of *C. militaris* are valuable for meeting increasing market demands. This is the first study, to the best of our knowledge, that utilizes the fluoride stimulation strategy for promoting the growth of *C. militaris*. Whereas high fluoride content (>1 mM) inhibited the formation of *C. militaris* fruiting bodies, the low level of fluoride (0.01 mM) promoted its growth and enhanced the content of bioactive substances including carotenoids and superoxide dismutase-like activity. Furthermore, extracts from FM *C. militaris* exhibited a stronger anti-proliferation effect on U2OS cancer cells.

## Figures and Tables

**Table 1:** Dry weight and total carotenoid content (TCC) of fruiting bodies from each medium.

*C. militaris* Samples	Dry Weight (g/bottle)	TCC (µg/g)
NM	1.07 ± 0.07	2561.27 ± 26.56
0.001 mM FM	1.16 ± 0.11	2751.00 ± 21.36
0.01 mM FM	1.55 ± 0.17	3161.38 ± 35.71
0.1 mM FM	1.28 ± 0.14	3035.96 ± 53.30

**Table 2:** Quantitation of fluoride in *C. militaris* fruiting bodies.

*C. militaris* Sample	Mass (x) g	n(std) mol	A(std)/A(x)	Fluorine (ppm)
0.001 mM FM	0.13	5.7 × 10^−4^	33,129.81	15.09
0.01 mM FM	0.09	5.7 × 10^−4^	21,350.02	33.81
0.1 mM FM	0.09	5.7 × 10^−4^	13,277.53	54.38

**Table 3:** 2,2-diphenyl-1-picrylhydrazyl (DPPH) radical scavenging activity and superoxide dismutase (SOD)-like activity of four fruiting body extracts.

*C. militaris* Sample	DPPH IC_50_ (mg/mL)	SOD IC_50_ (mg/mL)	SOD Vigor (U/mg)
NM	4.16	1.13	44.25
0.001 mM FM	2.92	0.81	61.37
0.01 mM FM	2.59	0.59	84.75
0.1 mM FM	2.89	0.87	57.47
